# Forecasting and prevention of water inrush during the excavation process of a diversion tunnel at the Jinping II Hydropower Station, China

**DOI:** 10.1186/s40064-016-2336-9

**Published:** 2016-05-23

**Authors:** Tian-xing Hou, Xing-guo Yang, Hui-ge Xing, Kang-xin Huang, Jia-wen Zhou

**Affiliations:** State Key Laboratory of Hydraulics and Mountain River Engineering, Sichuan University, Chengdu, 610065 Sichuan China; College of Water Resources and Hydropower, Sichuan University, Chengdu, 610065 Sichuan China; College of Architecture and Environment, Sichuan University, Chengdu, 610065 Sichuan China

**Keywords:** Water inrush, Diversion tunnel, Geological condition, Water inflow, Forecasting, Pressure head

## Abstract

**Introduction:**

Estimating groundwater inflow into a tunnel before and during the excavation process is an important task to ensure the safety and schedule during the underground construction process.

**Case description:**

Here we report a case of the forecasting and prevention of water inrush at the Jinping II Hydropower Station diversion tunnel groups during the excavation process. The diversion tunnel groups are located in mountains and valleys, and with high water pressure head. Three forecasting methods are used to predict the total water inflow of the #2 diversion tunnel. Furthermore, based on the accurate estimation of the water inrush around the tunnel working area, a theoretical method is presented to forecast the water inflow at the working area during the excavation process.

**Discussion and evaluation:**

The simulated results show that the total water flow is 1586.9, 1309.4 and 2070.2 m^3^/h using the Qshima method, Kostyakov method and Ochiai method, respectively. The Qshima method is the best one because it most closely matches the monitoring result. According to the huge water inflow into the #2 diversion tunnel, reasonable drainage measures are arranged to prevent the potential disaster of water inrush. The groundwater pressure head can be determined using the water flow velocity from the advancing holes; then, the groundwater pressure head can be used to predict the possible water inflow. The simulated results show that the groundwater pressure head and water inflow re stable and relatively small around the region of the intact rock mass, but there is a sudden change around the fault region with a large water inflow and groundwater pressure head. Different countermeasures are adopted to prevent water inrush disasters during the tunnel excavation process.

**Conclusion:**

Reasonable forecasting the characteristic parameters of water inrush is very useful for the formation of prevention and mitigation schemes during the tunnel excavation process.

## Background

During the construction of a tunnel, such as a high-speed railway tunnel or diversion tunnel, water inrush is one of the most common and complex geological disasters and has a large impact on the construction schedule and safety (e.g. Coli et al. 2008; Zarei et al. 2011). Furthermore, when serious water inrushes occur in tunnel construction, huge economic losses and casualties can occur. Because water inrush causes great harm to underground engineering, the prediction of the groundwater inflow into a tunnel is needed for designing the tunnel drainage system and for estimating the environmental impact of the drainage (e.g. Park et al. 2008; Wang et al. 2011. The prediction of water inflow into a tunnel involves two aspects: one is the total inflow prediction before excavation and the other is the estimation of the water flow at the working area during the excavation process. Forecasting the water inflow before excavation of a tunnel gives a rough estimate of the water inflow before tunnel construction. The prediction requires geological and hydrological parameters to be determined; then, formulas are used to calculate the water inflow. These forecasting methods for water inrush into a tunnel can be divided into roughly two categories: the water balance method and groundwater dynamics method (Zhu and Li 2000). The water balance method is based on the principle of water balance, and its calculated result is the average water inflow over a span of years. The groundwater dynamics method is based on the hydraulics principle and has wide applications (Schwarz et al. 2006).

Previous studies developed with several methods for forecasting water inflow during the tunnel excavation process. For example, Goodman (1965) proposed a relation between a homogeneous aquifer and an infinite water table. Li et al. (2009) presented a numerical method for forecasting the groundwater flow and distribution of pore water pressure around tunnels. Based on the well-known Jacob and Lohman (1952) solution, Marechal and Perrochet (2003) presented a theoretical model to forecast the transient ground water discharge into deep Alpine tunnels. El Tani (2003) presented an analytical solution of the groundwater inflow based on the Mobius transformation and Fourier series. Zhang and Franklin (1993) presented an analytical solution to predict the water flow rush into a rock tunnel using the hydraulic conductivity gradient. Kostyakov and Ochiai proposed two types of theoretical models to determine the stable water inflow in tunnels (Xu et al. 2005). In this paper, three forecasting methods based on groundwater dynamics theory are used to predict the total water inflow of the #2 diversion tunnel at the Jinping II Hydropower Station and are compared with the measured results to evaluate the forecasting method.

The problems encountered during the construction period are mainly related to the unexpected inflow of groundwater at some locations; predicting the location of water ingresses is often a difficult task (Huang and Lu 2007). To obtain a more accurate value of the inflow at a tunnel face or the area near it during tunnel excavation, the groundwater pressure in the tunnel working area needs to be determined. Then, the water inflow value can be accurately predicted using hydraulics theory. Groundwater pressure can usually be determined using seepage theory if the groundwater table and geological conditions are known which can indicate the head loss from water table to measuring point. Zhang (2006) presented a seepage load incremental theory for analyzing stress in a lining and its impact on the water inflow during the excavation process. Wang et al. (2008) proposed a theoretical model to estimate the water pressure on a lining under controlled drainage. Atkinson and Mair (1983), Shin et al. (2002), Yoo (2005) and Lee et al. (2007) draw the same conclusions using a numerical simulation. Other researchers focused on analytical solutions to calculate the pore water pressure to estimate the effective stress distribution at the tunnel perimeter (Fernández and Alvarez 1994). However, during the tunnel excavation process, the geological conditions are always unknown and change along the excavation axis, so it is hard to obtain an accurate loss of the pressure head. To overcome this problem, this paper presents a theoretical method to more accurately predict the groundwater pressure during the tunnel excavation process. First, the water inflow can be measured from an advanced borehole or grout-hole. Then, this value can be used to calculate the groundwater pressure through hydraulics theory and to forecast the water inrush that may occur at the tunnel working area so that suitable countermeasures can be presented.

## Project background

### Project overview

**Fig. 1:**
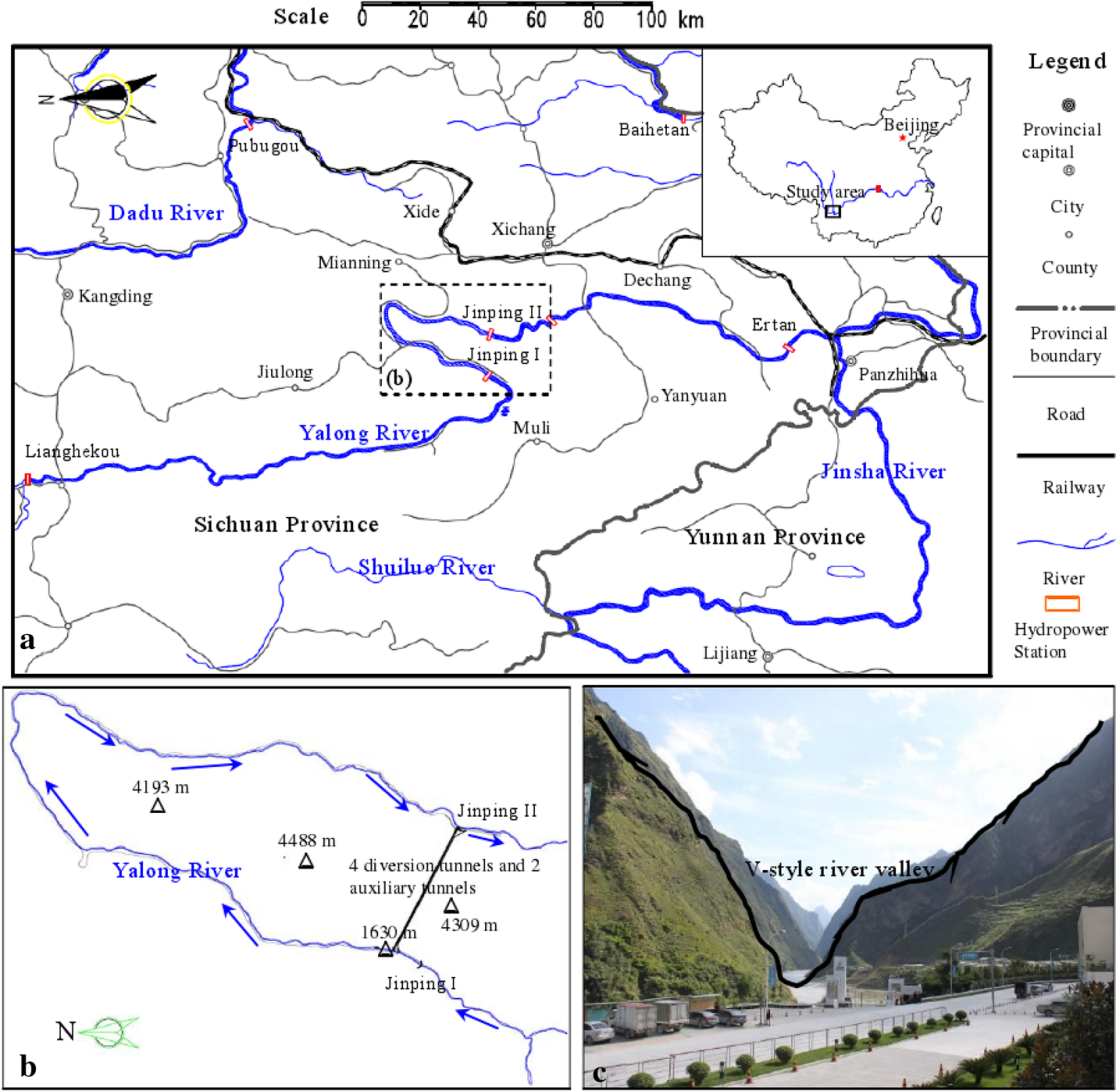
Jinping II Hydropower Station and its diversion tunnels group: **a** location of the Jinping I and II Hydropower Stations, **b** layout of the diversion tunnels group and **c** image of the dam site and tunnel intake

The Jinping II Hydropower Station is located at the convergence of Mili Tibetan Autonomous County, Yanyuan County and Mianning Country in Liangshan Yi Autonomous, Sichuan province, China (as shown in Fig. 1a). As a diversion-type hydropower station with a low brake, long tunnel, high pressure head and large capacity, it is an important cascade hydropower station along the Yalong River (Zhou et al. 2012). The powerhouse will use eight 600 MW turbine generators for a total generating capacity of 4800 MW (Xu and Shao 2009). The Jinping I Hydropower Station is situated upstream from the station, and the Guandi Hydropower Station and Ertan Hydropower Station are situated downstream from it. The 150 km natural drop of the bend downstream the Yalong River can produce a pressure head of approximately 310 m through a cutoff using the 16.67 km diversion tunnel, as shown in Fig. 1b.

**Fig. 2:**
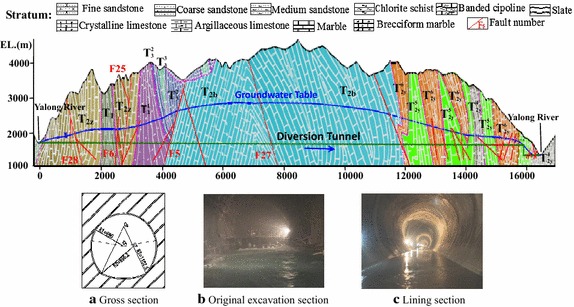
Geological condition of the longitudinal profile for the #2 diversion tunnel at the Jinping II Hydropower Station: **a** gross section; **b** original excavation section; and **c** lining section

The water diversion system of the Jinping II Hydropower Station adopts the layout with 4 tunnels and 8 sets across the Jinping Mountain, which is a typical V-style valley (as shown in Fig. 1c). The overlying rock mass generally has an embedded depth of 1500–2000 m along the diversion tunnel group, with a maximum depth of approximately 2525 m. Therefore, the water diversion system has a deep depth, long tunnel line and large diameter (Wu et al. 2008a). The Jinping II Hydropower Station tunnel group consists of four diversion tunnels, two auxiliary tunnels, and one drainage tunnel. The average length of the four parallel-arranged diversion tunnels is 16.67 km, and each of two adjacent tunnels are 60 m apart (Wu et al. 2008b; Shan 2009). The diversion tunnel group is located in the karst area of the high mountains and gorges with a complex geology (as shown in Fig. 2). According to the design report, except the exit sections of 1# and 3# diversion tunnels were excavated as a circle which excavated by TBM, other parts of diversion tunnels which by drilling and blasting are all excavated as a horseshoe-shaped section. Shown in Fig. 2a is the schematic diagram of cross section, and its real figures are followed by Fig. 2b, c. The main geological problems over this area are high ground stress, rock burst, water inrush, high ground temperature, harmful gases, stability of the surrounding rock, fault fracture zone through the tunnel (Wu et al. 2007).

### Geological condition

The area of the Jinping diversion tunnel groups are part of an alpine landscape with strong cutting structures and a large number of mountains with elevations of more than 3000 m. The direction of the Yalong River is approximately north-to-east 25° (N25°W), but when it arrives in the Heai country, it suddenly changes to south-to-west 15° (S15°W). Jinping Mountain, with an elevation of 4309 m, is located at the right bank of the Yalong River, and the valley is sharply incised by the river with an elevation less than 2000 m. In general, the terrain in the study area is very steep and the physiognomy mainly includes the following types: high mountains with strong cutting, mountains of medium cutting, gorges and karst and glacial geomorphology.

The Triassic strata is widely distributed in the research area, which accounting for more than 90 % of the area, and the outcropped area of carbonate rock makes up of 70–80 % among the area which is really an essential element of rock stability. Firstly, because of the strong extrusion in this kind of stratum, many complex folds had forming with the direction of SN. It has a significant impact on the water inrush because their cores and flanks can easily store water and form water channels. Then, the carbonate rock has an obvious solubility comparing with many other rocks, when it soaks in water for a long time, many karst caves may be form along the tunnel line and also brings some adverse effects for the diversion tunnels during the excavation process. Figure 2 shows the geological condition of the longitudinal profile for the #2 diversion tunnel at the Jinping II Hydropower Station. It shows the stratigraphic time from east to west includes: crystalline limestone, marble and argillaceous limestone in the Yantang Formation of the middle Triassic (T_2y_) which includes three rock formations (T_2y_^4^, T_2y_^5^, and T_2y_^6^); marble and crystalline limestone in the Baishan Formation of the middle Triassic (T_2b_); sandstone and slates in the upper Triassic (T_3_); crystalline limestone, marble, limestone, and argillaceous limestone in the Zagunao Formation of the middle Triassic (T_2z_); and chlorite schist, sandy mudstone, marble rocks in the Mojian Formation of the lower Triassic (T_1_).

As stated in the above paragraph, many folds has formed because of the strong extrusion in triassic strata. Based on the geological survey, a series of close complex folds have formed with a nearly north–south distribution and compression faults or compression-shear faults with a high dip angle in the study area, which is controlled by the tectonic stress field. Furthermore, some extensional faults and tension-torsional faults appear in this region. The folds in the study area are mainly compact folds, which include the Luoshuidong anticline, Jiefanggou compound syncline, Yangzhuchang compound syncline, Zumu anticline, Madang syncline and Dashuigou compound anticline. The structural surfaces in this area are mostly bedding extrusions or thrust faults, with large sizes and high frequencies. Faults mainly include the La Shagou-Yi Wanshui Fault, Jinping Mountain Fault and Shang Shoupa Fault. Joints and fissures developed in the area, especially at the folds and faults, except at those places with a thick and dense blocky rock mass. These geological structures have a great impact on the distribution of the groundwater in this region and affect the situation of the water inrush during the tunnel excavation process.

### Hydrological condition

**Fig. 3:**
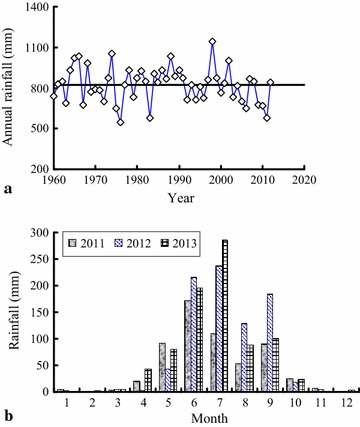
Rainfall data of the study area: **a** annual rainfall range from 1960 to 2012; **b** comparison of the monthly rainfall of 2011, 2012 and 2013

**Fig. 4:**
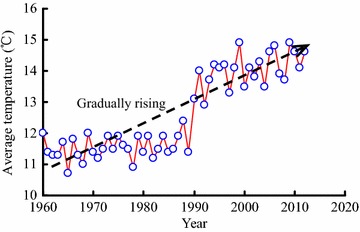
Annual average temperature of the study area range from 1960 to 2012

The Yalong River basin belongs to the climatic area of the western Sichuan plateau. Because the climate is mainly affected by the high-altitude west wind circulation and southwest monsoon, the wet and dry season are easily distinguished. Figure 3 shows the rainfall monitoring data of the study area. As shown in Fig. 3a, the annual rainfall is in a relatively stable range from the years of 1960 to 2012 (approximately 821.3 mm), so this can provide a stable water source for water inrush from rainfall. There is large rainfall from June to September (Fig. 3b), so water inrush is more serious during this time. For example, a series of water inrushes with high flow and pressure occurred in the research area on August 30, 2012. The annual average temperature has gradually risen from 1960 to 2012 in Mili Country (Fig. 4), which changed the annual evaporation from 1166 to 2500 mm.

According to the geological survey data of the study area, we can divide the aquifer group into the following types: pore aquifer rock group in the valley ground; fissure and karst-cave aquifer rock group in the carbonatite; and fissure aquifer rock group in the bedrock. Among them, the pore aquifer rock group in the valley ground is mainly distributed in the quaternary accumulation layer that is located in the terraces, slope toes and gentle slope zones; their main lithology includes gravel-cobble, silt, sandy clay, siltstone, gravel bed, and so on. The fissure and karst-cave aquifer rock group in the carbonatite is mainly distributed in the Baishan Formation of the middle Triassic (T_2b_) and Yantang Formation of the middle Triassic (T_2y_), and its lithology includes limestone, dolomite, marble, marlstone, and so on. The fissure aquifer rock group in the bedrock is mainly distributed in the Zagunao Formation (T_2z_), and its lithology includes meta-sandstone, slate, clasolite. A part of the groundwater recharged in the bedrock mountains of the higher ground has been discharged by runoff from high to low-lying areas. Another part is discharged into nearby valleys in the form of springs, where it will form an overflow area of the groundwater.

**Fig. 5:**
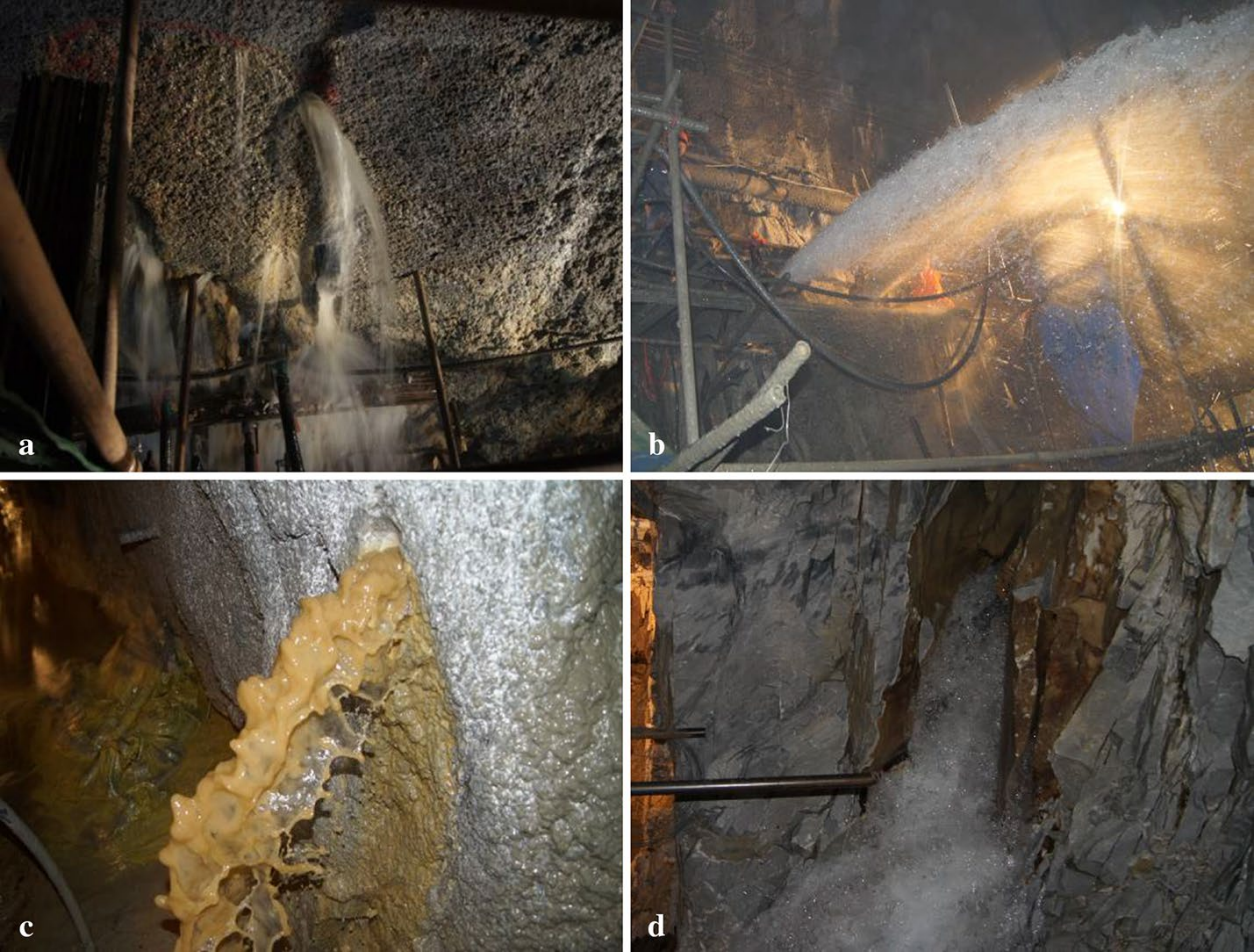
Water inrush disasters in the diversion tunnel during the construction process: **a** low flow water inrush; **b** large flow water inrush; **c** water inrush with silt; and **d** water inrush from a structural surface

The study area can be divided into three large hydrogeological units: an eastern independent hydrogeological unit, central hydrogeological unit and western hydrogeological unit. The karst development is not strong in the diversion tunnel zone, and the karst form mainly includes caverns and pipelines with no underground river or a large hall type karst. Because the development degree of karst in the central Jinping mountain is deeper than the east and west sides, it forms a series of springs that discharge at high elevations, such as the Laozhuangzi spring groups, Mofanggou spring, and Sangushui spring (Xu and Shao 2009). During the tunnel construction process, several water inrush disasters could occur in the diversion tunnels, which can be divided into different types. Figure 5 shows the typical water inrush disasters in the diversion tunnel during construction process, such as low flow water inrush in the joints or fractures (Fig. 5a), large flow water inrush in the faults with high pore water pressure (Fig. 5b), water inrush with silt in the fractures or faults (Fig. 5c) and water inrush with bubbles in the structural surfaces (Fig. 5d). Water inrush disasters have an adverse effect on the structural safety and construction progress, so accurately forecasting water inrush events and utilizing reasonable prevention methods during the tunnel excavation process are very important for tunnel construction.

## Total water inflow of the whole tunnel

For the water inrush in the #2 diversion tunnel at the Jinping II Hydropower Station, the total water inflow of the whole tunnel should be roughly estimated to formulate the overall drainage measures. Here, three different forecasting methods based on ground water dynamic theory are used to determine the total water inflow of the #2 diversion tunnel.

### Theoretical methods

**Fig. 6:**
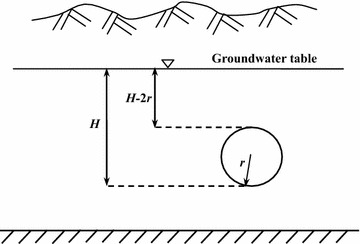
Calculation schematic of the groundwater dynamics method

Forecasting the water inflow into a tunnel is a hot issue in tunnel engineering because water inrush is one of the most serious disasters during the tunnel construction process. Theoretical formulas for the determination of water inflow in a tunnel can be roughly divided into two groups: the water balance method and groundwater dynamics method. The water balance method needs a series of extremely precise geological and hydrological parameters, but parameters along the tunnel line change greatly because of the complex geological and hydrological condition. Therefore, it is difficult to predict a value consistent with reality, so the water balance method can only be supplementary. In contrast, the groundwater dynamics method is based on generalized conditions and has widely applicability. This method treats the tunnel as an unlimited mostly confined aquifer with no water-resisting floor. Some empirical coefficients are added into the forecasting formulas that are derived from the basic principle of hydraulics, so it is a semi-empirical, semi-theoretical formula. As an extension of Goodman’s method, the calculation schematic plan of the groundwater dynamics methods is shown in Fig. 6. Here the Oshima method, Kostyakov method and Ochiai method are used to determine the total water flow of the whole #2 diversion tunnel as they are all based on the seepage theory but use different computing methods of seepage process to reflect the effect of underground water head on the water inflow into tunnels.

For the Oshima method, the water inflow per unit length can be calculated as follows (Zhang 2006):

1 $$q = \frac{{2\pi mk(H - r_{0} )}}{{\ln [2(H - r_{0} )/r_{0} ]}}$$

where *m* is a conversion coefficient; *k* is the permeability coefficient of the rock mass (m/d); *H* is the vertical distance from the groundwater table to the tunnel floor (m); and *r*_0_ is the equivalent radius of the tunnel cross section (m).

For the Kostyakov method, the water inflow per unit length can be determined as follows (Wu et al. 2007):

2 $$q = \frac{2akH}{\ln (R/r)}$$

where *R* is the influence radius of the tunnel drainage (m); a is a correlation coefficient; and *r* is half of the tunnel cross section width (m).

Water inflow per unit width using the Ochiai method can be expressed as follows (Wu et al. 2008b):

3 $$q = k\left[ {\frac{{H^{2} - h_{0}^{2} }}{R - r} + \frac{{\pi (H - h_{0} )}}{\ln (4R/W)}} \right]$$

where *h*_*0*_ is the water depth of the drainage ditch in the tunnel (m) and *W* is the tunnel cross section width (m).

### Parameters and simulated process

The geological condition along the tunnel is changeable. To obtain a relatively accurate value of the water inflow, the whole tunnel should be divided into different sections according to their hydrological and geological conditions. The #2 diversion tunnel is divided into ten sections, and Table 1 summarizes the engineering geological properties of the rock masses in the #2 diversion tunnel. The annual rainfall is 821.3 mm. Referring to the hydrogeological map of the diversion tunnel, the sum of the influence width from the two sides is *B* = 5 km. The equivalent radius of the tunnel cross section and half of the tunnel width are *r*_0_ = *r* = 6 m according to the design material. The water depth of the drainage ditch in the tunnel is *h*_*0*_ = 0. For the Kostyakov method, the influence radius of the tunnel drainage and correlation coefficient are estimated as $R = 2H\sqrt {kH}$ and *a* = π/2 + *H*/*R*. The conversion coefficient for using the Oshima method is *m* = 0.86.

**Table 1:** Engineering geological properties of the rock masses in the #2 diversion tunnel

No.	Tunnel section (m)	Stratum	Rock type	Pressure head (m)	Infiltration rate	Permeability (m/d)
1	K0 + 000 ~ 0 + 115	T_1_	Fine sandstone	76	0.15	0.008
2	K0 + 115 ~ 2 + 000	T_2z_	Marble	237	0.38	0.004
3	K2 + 000 ~ 2 + 500	T_1_	Chlorite schist	361	0.15	0.006
4	K2 + 500 ~ 3 + 316	T_2z_	Marble	401	0.38	0.004
5	K3 + 316 ~ 4 + 414	T_3_	Sandstone and slate	577	0.15	0.008
6	K4 + 414 ~ 8 + 265	T_2b_	Marble and Limestone	945	0.5	0.004
7	K8 + 265 ~ 12 + 571	T_2b_	Marble	876	0.38	0.004
8	K12 + 571 ~ 15 + 152	T_2y_	Marble and limestone	624	0.5	0.005
9	K15 + 152 ~ 16 + 151	T_2y_	Marble and slate	312	0.32	0.005
10	K16 + 151 ~ 17 + 291	T_2y_	Marble, limestone and slate	220	0.35	0.006

### Simulated results

**Fig. 7:**
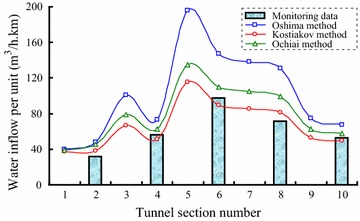
Simulated results for the water inflow per unit length using different forecasting methods based on the groundwater dynamics theory

Simulated results for the water inflow per unit length using different forecasting methods based on groundwater dynamics theory are shown in Fig. 7. As shown in Fig. 7, the evolution pattern of these three methods is basically the same. Their trends depend mostly on the vertical distance from the groundwater table to the tunnel floor. The water inflow rises as the distance between the groundwater table and the tunnel floor increases. The water flow per unit length is influenced by the geological and hydrological conditions. For example, the groundwater height at the fifth section (K3 + 316 ~ K4 + 414) is not the highest along the whole tunnel, but this section is full of fine sandstone and has a relatively high permeability coefficient, so the water inflow per unit length is the maximum value along the whole tunnel. However, the water flow has a lower value in the fourth section, which is full of marble with a low permeability coefficient. Beyond permeability, there are other factors that influence water inflow; several water inrush events in tunneling with large volumes of local groundwater inflows have occurred from geological features, such as fault zones, open fractures, and dykes (Tseng et al. 2001; Song et al. 2006). As the Oshima method adds a coefficient (*m*) into seepage formula to reflect the degree of reduction which can not easily work well for each case, the error between the forecasted value of water inflow per unit length using the Oshima method and monitoring data is the largest. But, while Kostyakov method and Ochiai method has used the seepage theory again to estimate the influence radius (*R*) of the tunnel drainage, they can work more accurately and the forecasted values are much closer to the monitoring data.

**Fig. 8:**
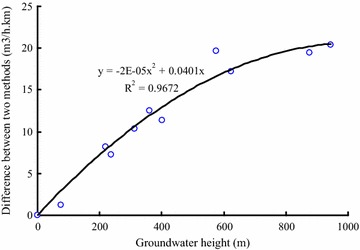
Difference between the Kostyakov and Ochiai methods versus the height of the groundwater table

Although the calculation results of the Kostyakov and Ochiai methods are very close, there still is some difference between these two methods. Figure 8 shows the difference between the Kostyakov and Ochiai method that vary with the height of the groundwater table, which exhibits a nonlinear trend. The difference between the Kostyakov and Ochiai methods increases as the groundwater table rises. The difference between the methods grows faster when the groundwater table is not very high than when the groundwater table is higher. Furthermore, the coefficient of permeability also has an effect on the variation trend of the curve in this figure. Obviously, the difference increases as the coefficient of permeability increases. The total water inflow of every section and the whole tunnel are summarized in Table 2. The total water inflow determined by the Kostyakov method is 1309.4 and 1586.9 m^3^/h by the Ochiai method. The possible maximum water inflow computed by the Qshima method is about 2070.2 m^3^/h. Water inflow during tunnel construction is not a constant process and will change with time as excavation continue, so there inevitably will be a maximum inflow during the process of water inrush. While Kostyakov method is also limited by the correlation coefficient (*a*), leading the computed result a little small, the simulated results show that the Ochiai method is the best method to forecast the steady total water inflow in tunnel engineering, and Oshima method is suited for estimating the maximum water inflow during the water inrush.

**Table 2:** Simulated result for the total water inflow of the whole tunnel using different forecasting methods

Tunnel section (m)	Stable water inflow (m^3^/h)		Maximum water inflow (m^3^/h)
	Kostyakov method	Ochiai method	Qshima method
K0 ~ 115	4.3	4.5	4.6
K115 ~ 2000	71.5	85.2	90.3
K2000 ~ 2500	33.3	39.6	50.2
K2500 ~ 3316	41.8	51.1	59.5
K3316 ~ 4414	126.5	148.0	215.1
K4414 ~ 8265	345.5	424.2	566.7
K8265 ~ 12571	366.6	450.5	595.0
K12571 ~ 15152	210.6	255.0	337.0
K15152 ~ 16151	52.6	62.9	74.4
K16151 ~ 17290	56.7	66.0	77.2
Sum	1309.4	1586.9	2070.2

### Engineering drainage measures

**Fig. 9:**
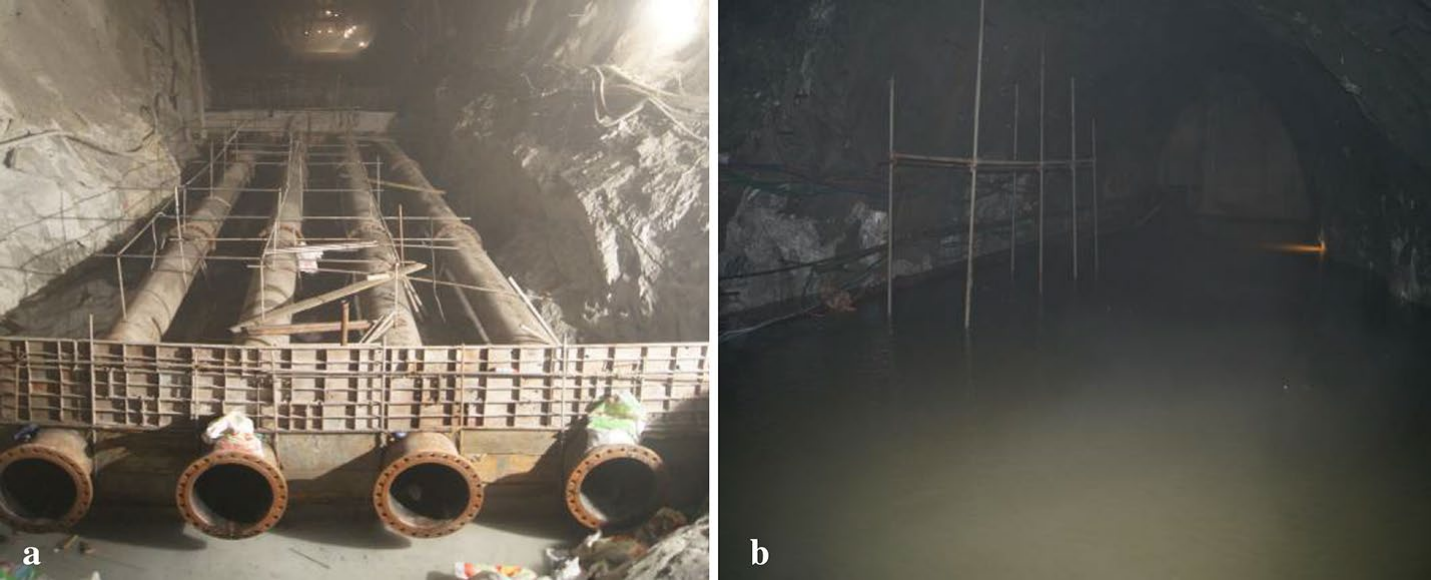
Drainage measures in the diversion tunnel: **a** installing of drainage pipe and **b** water catchment in the drainage tunnel before draining

The total water inflow of the whole tunnel directly affects the formulation of the overall drainage measures. As shown in Table 2, the total water inflow of the #2 diversion tunnel is very large and has a great impact on the safety and construction schedule. During the tunnel excavation process, field monitoring data shows that the total water inflow of the #2 diversion tunnel is approximately 1448.2 m^3^/h, which is much closer to the value forecasted by the Ochiai method. This value of total water inflow can be used to design the tunnel drainage system and engineering drainage measures. Because of the huge quantity of groundwater, the drainage tunnel and drainage pipes for water drainage must be large enough for the #2 diversion tunnel. Therefore, several water catchments are arranged along the tunnel axis direction (Fig. 9a). To drain the water out of the tunnel more effectively, galvanized steel pipes, with an inner diameter of 500 mm and thickness of 14 mm with no seams, were used in the #2 diversion tunnel. There were four parallel-arranged steel pipes used for the drainage system of the #2 diversion tunnel (Fig. 9b). According to the huge water inflow of the #2 diversion tunnel, a large number of drainage measures are arranged to prevent potential disasters caused by water flow.

## Water inrush around the tunnel working area

To forecast and prevent water inrush disasters during the tunnel construction process, an accurate estimation of the water inrush around the tunnel working area is critical for engineering and construction safety.

### Determination of the groundwater pressure head

**Fig. 10:**
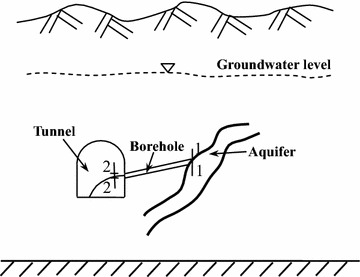
Generalized model for the determining the groundwater pressure around the tunnel working area

During the excavation process of the diversion tunnel, we must first measure the water rate from an advanced borehole to determine the groundwater conditions, such as the groundwater pressure head. The groundwater pressure head can be determined using the theory of hydraulics. Figure 10 shows the generalized model for the determination of the groundwater pressure around the tunnel working area.

Based on the Bernoulli equation (Wu et al. 2008a), for Sections 1-1 and 2-2, we obtain the following equation:

4 $$H_{1} = z_{1} + \frac{{P_{1} }}{\gamma } + \frac{{\alpha_{1} v_{1}^{2} }}{2g} = z_{2} + \frac{{P_{2} }}{\gamma } + \frac{{\alpha_{2} v_{2}^{2} }}{2g} + h_{w}$$

where z_1_ and z_2_ are the vertical distances from the center of Sections 1-1 and 2-2 to the base level (m); *P*_1_ and *P*_2_ are the pressures of two sections (Pa). Because Sections 2-2 contacts with the atmosphere, P_2_ is atmospheric pressure P_0_; *γ* is the bulk density of water (N/m^3^); *α*_1_ and *α*_2_ are the kinetic energy correction factors, taken as: *α*_1_ = *α*_2_ = 1; *h*_w_ is the pressure head loss (m); and *v*_1_ and *v*_2_ are the flow rates of the two sections (m/s).

Suppose the center of Sections 2-2 is located within the base level, which means z_2_ = 0. Then, Eq. (4) can be written as follows:

5 $$H_{1} = \frac{{P_{2} }}{\gamma } + z_{2} + \frac{{\alpha_{2} v_{2}^{2} }}{2g} + h_{w} = \frac{{P_{0} }}{\gamma } + \frac{{\alpha_{2} v_{2}^{2} }}{2g} + \xi \frac{{v_{2}^{2} }}{2g} + \lambda \frac{l}{d}\frac{{v_{2}^{2} }}{2g}$$

where *d* is the diameter of the hole (m); *l* is the length of the hole (m); *ξ* is the coefficient of the local pressure head loss; and *λ* is the coefficient of the processing pressure head loss.

Because the hole is cylindrical and its inlet is a right angle, we consider *ξ* to be 0.5. However, for *λ*, it is related to the flow state. When Re < 2000, the flow state is laminar flow and *λ* can be calculated by Eq. (6). When Re > 2000, we consider the flow to be turbulent flow. Calculation and experience show that almost all of the gushing water from a pipe is turbulent flow, so *λ* can be determined by the Kian method (Eq. 7) or pulsation theory formula of turbulent flow near a wall (Eq. 8).

6 $$\left\{ {\begin{array}{*{20}c} {\lambda = \frac{64}{\text{Re}}} \\ {\text{Re} = \frac{vd}{\upsilon }} \\ \end{array} } \right.$$

7 $$\lambda = \lambda \left( {\text{Re} ,\frac{\Delta }{d}} \right) = \frac{1.325}{{\left[ {\ln \left( {\frac{\Delta }{3.7d} + \frac{5.74}{{\text{Re}^{0.9} }}} \right)} \right]^{2} }}$$

8 $$\lambda = \lambda \left( {\text{Re} ,\frac{\Delta }{d}} \right) = 0.11\left( {\frac{\Delta }{d} + \frac{68}{\text{Re}}} \right)^{0.25}$$

where Re is the Reynolds number; *ν* is the kinematic viscosity (m^2^/s); and Δ is the roughness of the hole-wall (mm).

Thus, substitute Eq. (7) or Eq. (8) in Eq. (5), and then, obtain two equations to determine the pressure head:

9 $$H_{1} = \frac{{P_{0} }}{\gamma } + \left[ {\alpha_{2} + \xi + \frac{1.325}{{\left[ {\ln \left( {\frac{\Delta }{3.7d} + \frac{5.74}{{\text{Re}^{0.9} }}} \right)} \right]^{2} }}\frac{l}{d}} \right]\frac{{v_{2}^{2} }}{2g},\quad{\text{ method A}}$$

10 $$H_{1} = \frac{{P_{0} }}{\gamma } + \left[ {\alpha_{2} + \xi + 0.11\left( {\frac{\Delta }{d} + \frac{68}{\text{Re}}} \right)^{0.25} \frac{l}{d}} \right]\frac{{v_{2}^{2} }}{2g},\quad{\text{ method B}}$$

Finally, the pressure head can be determined by Eq. (9) or Eq. (10).

### Velocity of the water flow

Take the sections of K12 + 737 to K12 + 744 and K13 + 785 to K13 + 831 of the #2 diversion tunnel at the Jinping II Hydropower Station as examples. The geological survey result shows that the class of the surrounding rock of section K12 + 733 to K12 + 744 is mainly composed of macro-grained marble with a medium or thick layer. The surrounding rock of this section is relatively complete, so its permeability coefficient is low and water inrush is not serious. The geologic information of zone K13 + 785 to K13 + 831 shows that the surrounding rock is mainly composed of microcrystalline marble with a thin layer and that it also contains briquettes, development of bedding and calcite veins with a width of 1–3 cm. The main joint around this tunnel section is a flat and smooth fault, which is filled with a small amount of debris, iron-manganese materials, with a dip direction of 80°–100° and dip of 75–85°.

Use a drilling machine, such as a hydraulic down hole-drill, to drill holes at the tunnel face or the tunnel wall, a large quantity of water will flow out from the previously drilled holes. There is a different level of gushing water from the 20 grout holes for the section of K13 + 785 to K13 + 831 and 10 grout holes for the section of K12 + 737 to K12 + 744 before the process of water plugging and grouting. To overcome the difficulty of measuring the water flow, we stuck a mold bag plug in the hole and then measured the water storage per unit time by a graduated cylinder. The water flow velocity can be determined using the following equation:

11 $$v = \frac{4Q}{{\pi d^{2} }}$$

where *Q* is the water inflow of every borehole (m^3^/s) and *d* is the diameter of the borehole (m).

The water flow from each hole at these two sections is shown in Table 3 and Table 4, and the value of the flow velocities are also shown in these two tables. Assuming Δ = 0.2 mm and *d* = 75 mm, the groundwater pressure head at these two sections can be determined by Eq. (9) or Eq. (10).

**Table 3:** Computation results of the groundwater pressure head and flow velocity at the section of K12 + 737 to K12 + 744

No.	Grout hole	Length (m)	Water flow (L/s)	Flow velocity (m/s)	Reynolds number	Pressure head (m)	
						Method A	Method B
1	Y2DS-003-01	6	7.3	1.65	82,619	10.85	10.84
2	Y2DS-003-02	6	13.7	3.10	155,052	12.11	12.05
3	Y2DS-003-03	6	17.1	3.87	193,533	13.09	12.95
4	Y2DS-003-04	2	14.6	3.30	165,238	11.56	11.56
5	Y2DS-003-05	6	14.1	3.19	159,579	12.21	12.19
6	Y2DS-003-06	8	16.2	3.67	183,347	13.29	13.15
7	Y2DS-003-07	6	7.7	1.74	87,146	10.91	10.90
8	Y2DS-003-08	8	4.1	0.93	46,403	10.48	10.45
9	Y2DS-003-09	6	4.8	1.09	54,325	10.56	10.54
10	Y2DS-003-10	8	10	2.26	113,177	11.48	11.46

**Table 4:** Computation results of the groundwater pressure head and flow velocity at the section of K13 + 785 to K13 + 831

No.	Grout hole	Length (m)	Water flow (L/s)	Flow velocity (m/s)	Reynolds number	Pressure head (m)	
						Method A	Method B
1	Y2DS-001-01	8.00	39.20	8.87	443,654	27.40	27.22
2	Y2DS-001-02	8.00	55.90	12.65	632,659	44.93	43.95
3	Y2DS-001-03	8.00	55.10	12.47	623,605	44.95	42.63
4	Y2DS-001-04	8.00	46.10	10.43	521,746	33.90	33.66
5	Y2DS-001-05	8.00	6.20	1.40	70,170	10.78	10.78
6	Y2DS-001-06	8.00	6.20	1.40	70,170	10.78	10.78
7	Y2DS-001-07	8.00	18.40	4.16	208,246	14.14	14.09
8	Y2DS-001-08	8.00	37.59	8.51	425,432	26.03	24.87
9	Y2DS-001-09	8.00	0.20	0.05	2264	10.34	10.34
10	Y2DS-001-10	8.00	0.60	0.14	6791	10.34	10.34
11	Y2DS-001-11	8.00	197.50	44.70	2,235,245	439.74	427.93
12	Y2DS-001-12	8.00	179.40	40.61	2,030,394	364.73	354.56
13	Y2DS-001-13	8.00	10.24	2.32	115,893	11.53	11.52
14	Y2DS-001-14	8.00	0.34	0.08	3848	10.34	10.34
15	Y2DS-001-15	5.00	1.08	0.24	12,223	10.35	10.35
16	Y2DS-001-16	5.00	1.45	0.33	16,411	10.36	10.36
17	Y2DS-001-17	4.00	0.26	0.06	2943	10.34	10.34
18	Y2DS-001-18	4.00	0.62	0.14	7017	10.34	10.34
19	Y2DS-001-19	4.00	0.52	0.12	5885	10.34	10.34
20	Y2DS-001-20	4.00	0.52	0.12	5885	10.34	10.34

### Computational results

**Fig. 11:**
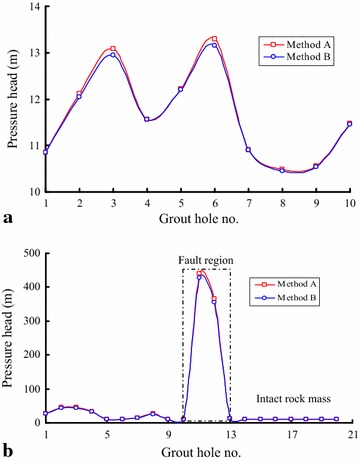
Simulated results of the groundwater pressure head at different locations: **a** section of K12 + 737 to K12 + 744; and **b** section of K13 + 785 to K13 + 831

Figure 11 shows the simulated results of the pressure head at different locations. For the section of K12 + 737 to K12 + 744, the pressure head determined by these two methods are all between 10 m and 13 m (Fig. 11a). Compared with the geological section map of this section, the geological condition around this region is mainly composed of intact rock masses, and there is no obvious development of geological structure in this region. During the excavation process of this section, a large water inrush will be unlikely. However, for the section of K13 + 785 to K13 + 831, the simulated results show that the pressure head at this section is extremely unstable and mainly between 10 and 45 m (Fig. 11b). There is a sudden change at the grout hole of 11 and 12, and the pressure heads for grout hole 11 and 12 are approximately 433 and 360 m. The main rock mass in this section is mainly composed by T6 2y, and a larger fault exists in this region. The pressure head sharply increased at the tunnel working area and resulted in a serious water inrush that affected construction safety.

The water inflow from the grout-hole at the section of K12 + 737 to K12 + 744 is low and also relatively stable; the maximum difference of the pressure head values calculated by the two methods is only 0.14 m. However, at the section of K13 + 785 to K13 + 831, the water inflow is extremely unstable and the maximum is 197.5 L/s, while the minimum is only 0.2 L/s. The difference of water pressure head values between the two methods is 11.81 m with a maximum water inflow and 0 for the minimum. From this analysis, we find that the value of water inflow from a borehole is a key factor in the difference between method A and method B. In other words, when the water flow is relatively fast, the difference is already relatively large, and when the water flows slowly, the difference is low as well.

### Countermeasures for water inrush

**Fig. 12:**
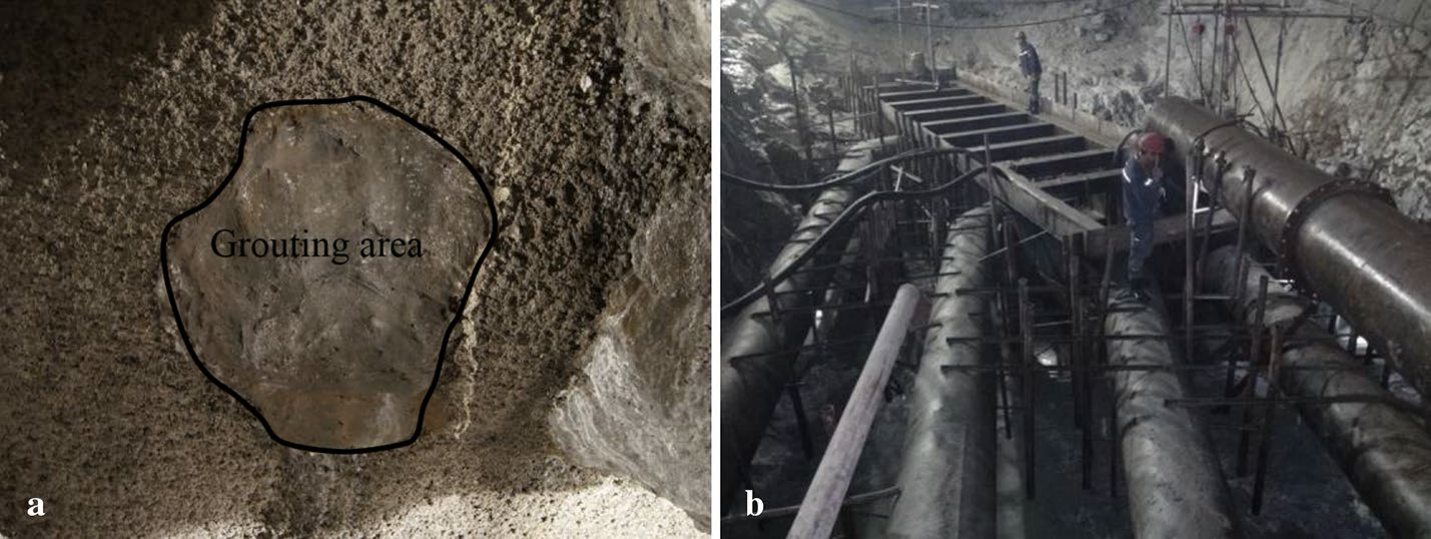
Groundwater plugging measures at water inrush points: **a** grouting technology for plugging; **b** caisson technology for groundwater treatment

The above computational results show that the maximum pressure head at the section of K12 + 737 to K12 + 744 is 13 m and section of K13 + 785 to K13 + 831 is 434 m. Because the groundwater pressure head is known in the execution area, we can predict the possible water inflow if water inrush occurs at the tunnel working area or a tunnel wall in a nearby area. Because an aquifer is always directly revealed during excavation, assume that the length of the hole is 0. Assuming a diameter of the flow hole revealed by excavation is 0.04 m^2^, the maximum water inflow at the two sections will reach 0.236 and 2.976 m^3^/s, respectively. For different types of water inrush at the working area, countermeasures for plugging the water flow are different (Fig. 12). Groundwater inflow is not very high at the section of K12 + 737 to K12 + 744, so just using simple grouting technology can plug the water (Fig. 12a). However, at the section of K13 + 785 to K13 + 831, the water inflow may be quite large, so only using grouting may not work. Therefore, a special caisson technology for groundwater treatment (Fig. 12b) in Jinping II Hydropower Station diversion tunnel must be used. This technology can gather the groundwater together into a caisson and drain it out by drainage pipes, which helps reduce the stress of the groundwater and ensure the structural security of the diversion tunnel as much as possible.

## Conclusions

The Jinping II Hydropower Station diversion tunnel groups located in mountains and valleys are very complex because of the geological and hydropower conditions, so it is inevitable that some significant water inrush accidents will occur during the excavation process. As a result, estimating the total water inflow before the excavation process is necessary to design the tunnel and its drainage system. Forecasting the water inrush at the working area is also important to determine the construction scheme.

In this paper, we used the groundwater dynamics method to predict the water inflow into the tunnel at every section. The calculated results are similar to the monitoring data. Through the comparison of the three types of groundwater dynamics methods, we conclude that the vertical distance from the groundwater level to the tunnel floor is the most important factor that affects the calculation. Among these three methods, the Kostyakov method and Ochiai method can forecast a relatively long-term and stable water flow into the tunnel, so they can be used to design the drainage system. The Oshima method can predict the maximum possible water inflow, so it can be used as a conservative value.

Water inrush from an actual tunnel face or its adjacent area only based on the above methods is difficult to forecast during excavating. To understand the groundwater condition fully and make an accurate prediction for the water inrush that may occur at the working area, this paper uses the hydraulics principle to calculate the pressure head on the basis of the water flow from a borehole; then, it ascertains the pressure head of different aquifers according to the geological section sketch map. Through this calculation, we can find the pressure head in an unconfined aquifer to be approximately 11 m in the section of K12 + 737 to K12 + 744 and between 10 and 45 m in the section of K13 + 785 to K13 + 831. Because there is a structural plane throughout the aquifer in the latter section, the pressure head in this structural plane is over 430 m. Therefore, a large water inrush point is most likely to be revealed if excavation continues, so more attention must be taken. To treat the groundwater more effectively, two different countermeasures (grouting technology and caisson technology) are needed to plug different water inrushes.
